# Identification of a novel MYO7A mutation in Usher syndrome type 1

**DOI:** 10.18632/oncotarget.23408

**Published:** 2017-12-19

**Authors:** Ling Cheng, Hongsong Yu, Yan Jiang, Juan He, Sisi Pu, Xin Li, Li Zhang

**Affiliations:** ^1^ The First Affiliated Hospital of Chongqing Medical University, Chongqing Key Laboratory of Ophthalmology and Chongqing Eye Institute, Chongqing, P. R. China; ^2^ Department of Ophthalmology, Yongchuan Hospital, Chongqing Medical University, Chongqing, P. R. China; ^3^ Department of Immunology, Special Key Laboratory of Gene Detection & Therapy of Guizhou Province, Zunyi Medical University, Guizhou, P. R. China

**Keywords:** Usher syndrome, USH1 family, *MYO7A*, novel mutation

## Abstract

Usher syndrome (USH) is an autosomal recessive disease characterized by deafness and retinitis pigmentosa. In view of the high phenotypic and genetic heterogeneity in USH, performing genetic screening with traditional methods is impractical. In the present study, we carried out targeted next-generation sequencing (NGS) to uncover the underlying gene in an USH family (2 USH patients and 15 unaffected relatives). One hundred and thirty-five genes associated with inherited retinal degeneration were selected for deep exome sequencing. Subsequently, variant analysis, Sanger validation and segregation tests were utilized to identify the disease-causing mutations in this family. All affected individuals had a classic USH type I (USH1) phenotype which included deafness, vestibular dysfunction and retinitis pigmentosa. Targeted NGS and Sanger sequencing validation suggested that USH1 patients carried an unreported splice site mutation, c.5168+1G>A, as a compound heterozygous mutation with c.6070C>T (p.R2024X) in the MYO7A gene. A functional study revealed decreased expression of the *MYO7A* gene in the individuals carrying heterozygous mutations. In conclusion, targeted next-generation sequencing provided a comprehensive and efficient diagnosis for USH1. This study revealed the genetic defects in the MYO7A gene and expanded the spectrum of clinical phenotypes associated with USH1 mutations.

## INTRODUCTION

Usher syndrome (USH) is recognized as the most common form of hereditary deaf-blindness [1]. USH is an autosomal recessive disease that is characterized by clinical and genetic heterogeneity. The frequency of USH has been reported to range from 3.8 to 6.2/100 000 in different countries, such as Spain, Denmark and the United States [2–4]. The prevalence of USH in China has not been reported. USH can be subdivided into the 3 following clinical types: USH type I (USH1), USH type II (USH2), and USH type III (USH3). The classification of USH is mainly based on the severity and progression of hearing loss (HL), the status of vestibular function and age at onset of retinitis pigmentosa (RP). Patients with USH1 typically have profound and congenital HL, which is further accompanied by vestibular areflexia and progressive RP of prepubertal onset. Patients with USH2 present with moderate-to-severe HL with down-sloping pure tone audiograms, normal vestibular function, and RP in adolescence. USH3 is very rare and variable in presentation. It may resemble USH1 or USH2, and vestibular dysfunction may be present [5].

For USH1, at least seven different loci can be mapped (USH1B-USH1H), but only the five following genes have been isolated: myosin VIIa (*MYO7A*), cadherin-23 (*CDH23*), protocadherin-15 (*PCDH15*), harmonin (*USH1C*), and SANS (*USH1G*) [6, 7]. A previous study has reported that USH1B is the most common form of USHI and is responsible for approximately 70% of all cases [8]. Meanwhile, *MYO7A* is mainly responsible for USH1B and accounts for approximately 50% of all pathogenic mutations [9, 10], followed by four other mutated genes (*CDH23*, *PCDH15*, *USH1C*, and *USH1G)*. The *MYO7A* gene is located on the long arm of chromosome 11 at position 13.5 (11q13.5). Human *MYO7A* comprises 49 exons spanning approximately 120 kb of genomic DNA. *MYO7A* codes for a 2215 amino acid protein, myosin VIIa, which has a molecular mass of 254 kDa [11]. Defects in *MYO7A* result in a wide phenotypic spectrum, including RP, sensorineural hearing impairment and vestibular dysfunction [12–14]. Most of these *MYO7A* mutations (more than 95%) cause Usher syndrome type 1. Successful identification of USH1 causative mutation provides more probability for elucidating the underlying pathophysiology of USH.

Compared with traditional approaches including direct sequencing and linkage disequilibrium, next-generation sequencing (NGS) has been demonstrated as a significant improvement that provides precise diagnostic information and extends the possibility of targeted treatments. Recently, studies have confirmed that specific genes (*TMC1, PCDH15, MYO7A* and *MYO15A*) were associated with hereditary HL in families by the NGS method [15]. Furthermore, NGS techniques have also been applied to identify 100 retinal disease genes, resulting in a higher detection rate of approximately 50% for RP cases [16–18]. Additionally, NGS is much faster, more cost-efficient and more accurate than traditional methods [19, 20]. In this study, a mutation screening of 135 candidate genes associated with inherited retinal degeneration, including RP and USH, was carried out on a proband using targeted NGS. We identified a novel *MYO7A* splicing site mutation (c.5168+1G>A) in a Chinese USH1 family, and found that the compound heterozygous mutations c.6070C>T (p.R2024X) and c.5168+1G>A in the *MYO7A* gene could lead to USH1.

## RESULTS

### Clinical phenotype of the Chinese Usher syndrome pedigree

**Figure 1:**
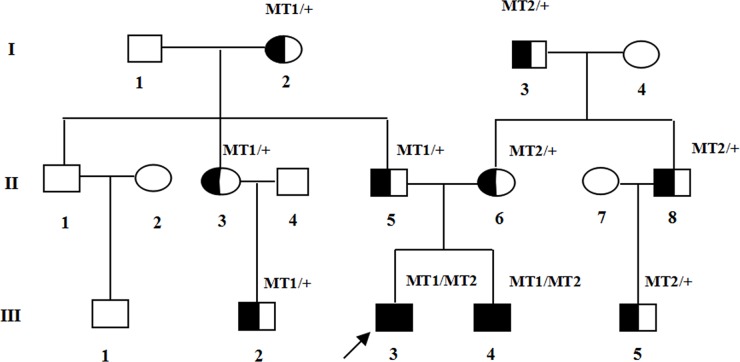
Pedigree of the family with USH1 Normal males and female are shown with empty squares and circle, respectively. Affected patient is shown with filled symbols. The proband (III:3) is indicated by arrow. MT: mutant type. MT1: c.5168+1G>A; MT2: c.6070C>T (p.R2024X).

**Figure 2:**
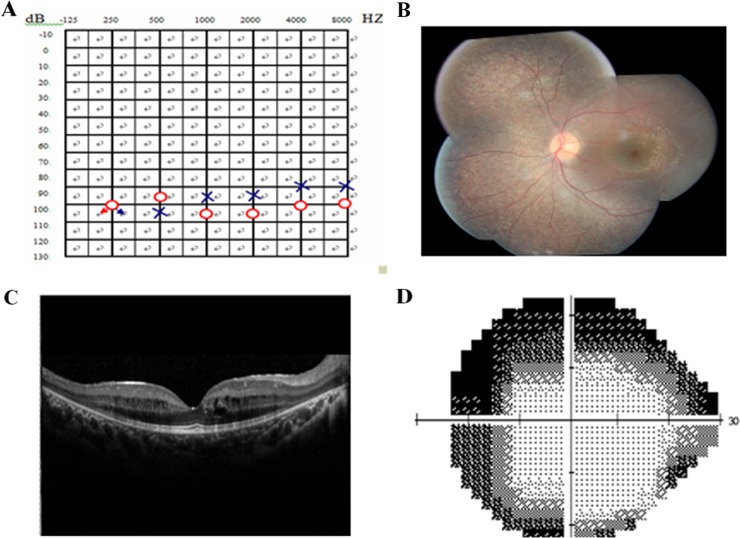
Pure tone audiogram, fundus photography, optical coherence tomography (OCT), and visual field of the proband **(A)** Audiogram showed bilateral profound sensorineural hearing loss of proband III: 4(red, right ear; blue, left ear). **(B)** Fundus photography showed atrophy of the retinal pigment epithelium, attenuation of retinal arterioles, pallor of optic disc and bone-spicule pigmentation in the retinal periphery. **(C)** OCT revealed cystoid macular oedema, macular retinoschisis and thinned retina in proband. **(D)** Bilateral vision field got concentric narrowing, for the residual central visual field were 10-20 degrees. The extent of visual field ranged from 10° to 20°.

Two affected members and fifteen unaffected individuals in an USH1 family were enrolled in this study. In this family, the proband had an affected brother, while the parents had no symptoms or signs of USH1 (Figure 1). The affected individuals included in the study suffered from vestibular dysfunction manifested by delayed walking (after two years old). All affected individuals showed profound bilateral sensorineural hearing impairment with vestibular response according to their pure tone audiometry results (Figure 2A). Next, detailed ophthalmological examinations were conducted. Fundus photography showed atrophy of the retinal pigment epithelium, attenuated arteries, pallor of the optic disc, bone-spicule pigmentation in the retinal periphery, and all typical signs related to RP (Figure 2B). The optical coherence tomography (OCT) presentations of proband III:3 and proband III:4 individuals exhibited cystoid macular edema, macular retinoschisis and retinal thinning (Figure 2C). Visual fields were restricted, ranging from 10° to 20° (Figure 2D). The best correct visual acuity (BCVA) of the two affected individuals ranged from 0.4 to 0.8. According to the typical clinical presentation and results from the audiometric test and ophthalmic examinations, we concluded that the proband and his older brother exhibited autosomal recessive USH1.

### Targeted exome sequencing identified candidate mutations

Targeted exome sequencing was performed for the proband sample from the USH1 family. The average depth of the targeted regions was at least 100-fold, with an overall coverage of the targeted regions equal to 98.0% for 10 or more reads. The Burrows-Wheeler Aligner (BWA) program was used to process raw data, and numerous non-synonymous variants were identified by using the genome analysis toolkit (GATK) program. The most likely pathogenic mutations were identified through the following approaches: 1) Functional variants (insertion/deletion: in coding sequence and splicing region, SNP: nonsense, splice site and missense). 2) The allele frequency of variants residing around or under 0.01 in any of the abovementioned databases. 3) Pathogenicity predictions with three computational tools were conducted; if at least one of the programs predicted the variant to be possibly damaging, it was considered pathogenic. Finally, using the stepwise approach as described, only two compound heterozygous mutations in *MYO7A* were identified in this pedigree (c.6070C>T (p.R2024X) and c.5168+1G>A).

### Validation of the mutations by sanger sequencing confirmation

The NGS results of the sample from the proband (III:2) showed probable defects in the *MYO7A* gene. We performed Sanger sequencing to validate the two heterozygous variants in *MYO7A* that were identified in the proband. Meanwhile, Sanger sequencing was also used to screen the same mutation sites in unaffected family members and the affected sibling. We confirmed two compound heterozygous mutations in this USH1 family, and one mutation in *MYO7A* has not been reported in any of the four SNP databases (dbSNP (Build 138), HapMap database, 1000 Genome Project and a local control database).

**Figure 3:**
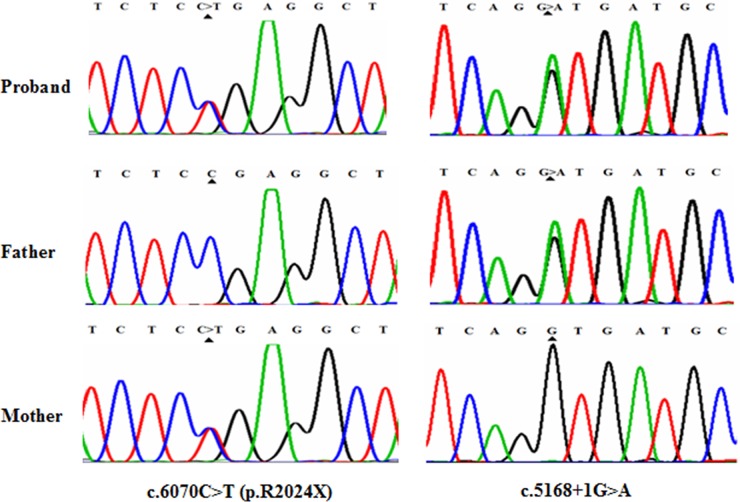
Mutational analysis of MYO7A in USHI family DNA sequencing profile showing the c.6070C>T (p.R2024X) and c.5168+1G>A mutations in MYO7A. Both variants co-segregated with the clinical phenotype.

The proband and the affected sibling harbored compound heterozygous *MYO7A* mutations c.6070C>T (p.R2024X) and c.5168+1G>A from the parents. c.5168+1G>A, a heterozygous splice site mutation (Figure 3), was found in exon 37 of *MYO7A* in the proband (III:3) and also in his affected sibling (III:4), unaffected father (II:5), aunt (II:3), cousin (III:2) and unaffected grandmother (I:2). Another mutation, c.6070C>T (p.R2024X), a nonsense mutation (Figure 3), was identified in exon 45 of *MYO7A* in the proband (III:3) and also in his affected sibling (III:4), his mother (II:6), uncle (II:8), cousin (III:5) and his maternal grandfather (I:3). Mutation (c.5168+1G>A), confirmed by Sanger sequencing, was inherited from the grandmother (I:2), and (c.6070C>T (p.R2024X)) was inherited from the maternal grandfather (I:3).

### Analysis of MYO7A mutations

Mutation c.6070C>T (p.R2024X), a substitution of C to T in exon 45 of the MYO7A gene has been reported in an earlier study [21]. This substitution is a nonsense variant predicted to alter an arginine into a stop codon at amino acid position 2024 of the myosin VIIa protein (total length: 2215 amino acids). Mutation p.R2024X might lead to dysfunction or reduction of myosin VIIa through truncation of the generated protein or nonsense-mediated mRNA decay (NMD), respectively. c.5168+1G>A, a splicing site mutation, has not been reported in earlier studies or in any other SNP database. Mutation c.5168+1G>A was a change from G to A at the conserved donor splice site in exon 37. According to online splice site prediction software (splice), mutation c.5168+1G>A was predicted to abolish the splice donor site, resulting in a premature termination codon (PTC) located at c.5168+32 to c.5168+34 in exon 37, TAG, leading to NMD and/or the generation of a truncated protein. The two compound heterozygous mutations c.6070C>T (p.R2024X) and c.5168+1G>A in *MYO7A* were highly conserved (GERP++ scores of 3.70 and 3.92, respectively). In summary, we successfully identified two truncating variants that were predicted to introduce a premature stop codon and therefore predicted to disrupt gene and protein function. The two mutations detected with NGS were verified by Sanger sequencing. Familial segregation analyses were also performed by polymerase chain reaction (PCR) and Sanger sequencing (Figure 1).

### Analysis of mutation pathogenicity in gene *MYO7A*

**Figure 4:**
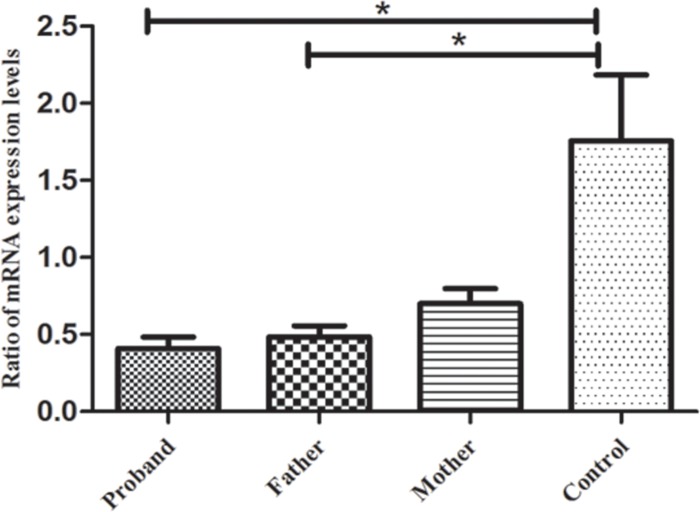
RT-PCR results for gene MYO7A The results indicated that the mRNA expression levels of gene MYO7A in the proband and her father were significantly lower than those of the normal controls (*p* < 0.05), but no significant difference was found between the proband's mother and the normal controls (*p* > 0.05). ^*^p < *0.05*, statistical significance was determined with a Student's *t* test.

The aforementioned result showed two compound heterozygous mutations in this USH1 family. Mutation c.6070C>T (p.R2024X), reported in an earlier study, is a nonsense mutation, whereas mutation c.5168+1G>A is a novel mutation, which could potentially affect a consensus donor splice site essential for splicing of exon 37. To assess the possible effect of the mutations on the expression of *MYO7A,* RT-PCR was performed to analyze the total lymphocyte RNA obtained from the affected sibling and the parents. The results showed that premature stop codons, which were predicted to create two mutations, apparently activate the NMD response, resulting in decreased *MYO7A* mRNA expression (Figure 4, P < 0.05). The two predicted compound heterozygous mutations can cause complete dysfunction of myosin VIIa in the same subject and lead to the observed phenotype in both the proband and affected sibling.

## DISCUSSION

In this Chinese USH1 family, we identified one reported mutation (c.6070C>T (p.R2024X)) and one novel splice mutation (c.5168+1G>A) using NGS methods. Both the proband and his sibling carried compound heterozygous mutations in *MYO7A* (c.5168+1G>A and c.6070C>T (p.R2024X)). These mutations were considered pathogenic mainly due to the following reasons: 1) mutation co-segregation in this family; 2) pathogenicity predictions with three computational tools (Mutation Taster, PolyPhen-2 and Sorting Intolerant From Tolerant (SIFT); and 3) the mutations were not seen in the 1000-Genomes Project or any other single nucleotide polymorphism database.

**Figure 5:**
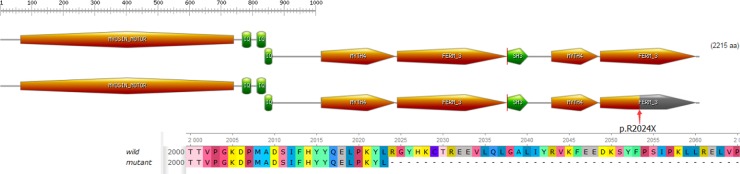
Domain structure of myosin VIIa The nonsense mutation introduces a premature stop codon which is predicted to truncate the protein within the N-terminal motor domain.

The protein myosin VIIa contains three typical domains (Figure 5): an N-terminal head domain (1-729) or motor, a neck region containing five isoleucine glutamine motifs (IQ) (IQ1-IQ5: 745-857), and a tail beginning with a short coiled-coil region domain for dimerization (858-935), followed by two MyTH4-FERM repeat elements (1017-1602) (1747–2205), separated by an SH3 domain (1603–1672) [22]. Myosin VIIa plays an important role in the retinal pigment epithelium and photoreceptor cells, as well as in the inner ear, as a type of molecular motor. In photoreceptor cells, myosin VIIa displays an active function in the transport of opsin from the inner segment to the outer segment of photoreceptors through the connecting cilium [23]. In the human retina, myosin VIIa functions actively in the migration of RPE melanosomes, phagocytosis of photoreceptor cell outer segment tips and opsin transport through the cilium of these photoreceptors [24, 25]. The nonsense mutation c.6070C>T (p.R2024X) and the splice site mutation c.5168+1G>A may cause complete functional loss of myosin VIIa protein. The nonsense mutation, c.6070C>T (p.R2024X), can generate a PTC, containing only 2024 amino acids with truncation of the last 191 amino acids (Figure 5). This mutant protein lacks the tail region. The tail domain of MYO7A is believed to be largely responsible for its function [26]. Mutation c.5168+1G>A is predicted to lead to a PTC (TAG, located at c.5168+32 to c.5168+34 in exon 37) after a G to A transition at the splice acceptor site. As a result, mutation c.5168+1G>A may be involved in abnormal RNA processing and trigger a truncated MYO7A protein. Both identified compound heterozygous mutations could lead to a truncated protein that disrupts the normal function of myosin VIIa, which can lead to the observed phenotype of the proband and the affected sibling.

For hereditary diseases including USH1, comprehensive molecular testing can help to elucidate the molecular pathogenesis, provide early diagnosis, and explain the severe clinically observed symptoms. Using targeted NGS, we can fulfill ethnicity-specific diagnosis of USH. However, our study has several limitations. First, NGS is not a routine application that can be used in clinic because of the cost, infrastructural logistics, strategies for patient selection, and interpretation of genetic datasets [27]. In addition, compared with whole genome sequencing, our approach detected only 135 genes related to inherited retinal degeneration. NGS technology cannot identify mutations in deep intronic regions, structural variations or large copy-number. Furthermore, members in this family with both mutations had severe symptoms and signs of USH1, while others with one or without mutations were completely normal. These phenotypic differences might be partially due to the complex interactions between the two mutations. Therefore, we should perform additional functional studies to determine the complex relationship between the two mutations and the pathogenesis of distinct mutations.

In conclusion, we determined that the compound heterozygous mutations, c.6070C>T (p.R2024X) and c.5168+1G>A, could result in USH1 in a Chinese family. Our finding expands the spectrum of clinical phenotypes of mutations, which can help us better understand the genotype-phenotype relationship in this disease, and provides the potential for early molecular diagnoses, accurate genetic counseling, and optimal rehabilitation.

## MATERIALS AND METHODS

### Subjects

A three-generation consanguineous Chinese family diagnosed with USH1 was enrolled in this study. The pedigree of the family contains 17 members and is shown in Figure 1. A full medical and family history was recorded for all family members, mainly including intelligence and growth development, hearing impairment and visual loss. A routine ophthalmologic examination, including BCVA, intraocular pressure, slit-lamp biomicroscopy, and dilated indirect ophthalmoscopy, was performed in the department of ophthalmology at the First Affiliated Hospital of Chongqing Medical University (Chongqing, China). The proband and affected patient underwent an additional eye examination, including visual field, fundus photography, OCT, visual evoked potential, synoptophore and visual function examination. An otology examination was also performed, including audiometric examinations and vestibular evaluation by otolaryngologists. All participating individuals provided written informed consent before donating venous blood. This study was approved by the Local Ethics Research Council and was conducted according to the principles of the Declaration of Helsinki. All experiments were conducted in accordance with the approved guidelines and regulations.

### Targeted region capture and NGS

A total of 135 inherited retinal degeneration-related genes (Supplementary Table 1) were selected using a solution-based sequence capture custom enrichment kit (GenCap, MyGenostics, Beijing, China). Peripheral blood samples were collected from the proband, and genomic DNA extraction was performed using a QIAamp DNA Blood Mini Kit (Qiagen, Valencia, CA, USA). The quantity and quality of the DNA were verified using a NanoDrop 2000 (Thermo Fisher Scientific, Delaware, USA). Next, genomic DNA (1 μg) was fragmented into 150-300 bp by a Covaris sonicator (Covaris S2, Massachusetts, USA). After end-repair and adapter ligation, the fragments underwent 4 cycles of PCR amplification with Illumina PCR primers. The purified PCR products (500 ng) were hybridized to the GenCapTM probe (in solution) for 16 hours. After hybridization, washing, and elution, the eluted fraction underwent 14 cycles of PCR amplification and was subsequently subjected to Qubit and quantitative PCR to estimate the magnitude of enrichment. Each eluted, enriched DNA sample was then sequenced on HiSeq2000 Analyzers (Illumina, San Diego, USA) for 90-bp paired-read sequencing, providing an average coverage depth of each sample of at least 100-fold. Image analysis and base calling was performed using Illumina Pipeline (version 1.3.4) with default parameters.

### Bioinformatics analysis

Bioinformatics analysis was conducted as previously described [28]. To identify the underlying variants of the proband, the 90-bp clean reads were subjected to alignment with the reference human genome (UCSC hg19) database (http://hgdownload.cse.ucsc.edu/goldenPath/hg19/bigZips/) using the BWA Multi-Vision software package (http://bio-bwa.sourceforge.net/) [29]. Next, single and multiple nucleotide variants, including small insertion or deletions, were identified by GATK software [30]. Several common variant databases, such as the 1000 Genomes database (http://www.1000genomes.org/), dbSNP (Build 138) (NCBI, http://www.ncbi.nlm.nih.gov/SNP/ [in the public domain]), HapMap database (ftp://ftp.ncbi.nlm.nih.gov/hapmap), and a local control database, were used to remove all variants with an allele frequency above 1%.

### Mutation validation and silico analyses

After filtration, variants were subsequently verified by PCR and Sanger sequencing of samples from the proband and family members. Peripheral blood samples from the family members were collected in ethylenediaminetetraacetic acid (EDTA) anti-coagulated tubes and stored at -80°C until used. Genomic DNA was extracted by a QIAamp DNA Blood Mini Kit (Qiagen, Valencia, CA, USA) according to the manufacturer's instructions. Sanger sequencing was applied to perform segregation tests for all participants. Amplification of the target DNA sequence in the *MYO7A* gene was analyzed by PCR using the primers presented in Supplementary Table 2. Three online software programs, Polymorphism Phenotyping (PolyPhen-2, http://genetics.bwh.harvard.edu/pph2/), SIFT (http://sift.bii.a-star.edu.sg/), and Mutation Taster (http://www.mutationtaster.org), were used to evaluate the possible pathogenic effects of the mutations.

### Real-time PCR

Total RNA was isolated from peripheral blood mononuclear cells (PBMC), which were obtained from the USH1 family members, with TRIzol (Invitrogen, San Diego, California, USA). Reverse transcription was performed by a transcriptase kit (Takara, Dalian, China). Relative mRNA expression was measured by a 7500 Real-Time PCR Instrument (ABI). MYO7A and β-actin expression levels were tested by the following primers (*MYO7A*, forward primer: 5’-CGCTACCGGGACCACCTC-3’ and reverse primer: 5’-GGGCATCTCCCCTATCTTC-3’; β-actin, forward primer: 5′-GGATGCAGAAGGAGATCACTG-3′ and reverse primer: 5′-CGATCCACACGGAGTACTT-3′). The relative expression level of MYO7A was computed using the 2^-ΔΔCt^ method.

## SUPPLEMENTARY MATERIALS FIGURES AND TABLES


