# Supplementary material for: Identification of a novel MYO7A mutation in Usher syndrome type 1
Source: Oncotarget. 2017 Dec 19;9(2):2295–303. doi: 10.18632/oncotarget.23408 (PMC5788640; doi:10.18632/oncotarget.23408)
Supplement: Supplementary file 1 [file oncotarget-09-2295-s001.pdf]

Identification of a novel MYO7A mutation in Usher syndrome type 1

SUPPLEMENTARY MATERIALS

Supplementary Table 1: A total of 135 inherited retinal degeneration related genes investigated in this study

No.	Gene	No.	Gene	No.	Gene	No.	Gene	No.	Gene
1	*ABCA4*	28	*CEP290*	55	*IDH3B*	82	*PDE6A*	109	*RPGRIP1L*
2	*ABHD12*	29	*CEP41*	56	*IFT27*	83	*PDE6B*	110	*SAG*
3	*AHI1*	30	*CERKL*	57	*IMPDH1*	84	*PDE6D*	111	*SDCCAG8*
4	*AIPL1*	31	*CIB2*	58	*IMPG2*	85	*PDE6G*	112	*SEMA4A*
5	*ARL13B*	32	*CISD2*	59	*INPP5E*	86	*PDZD7*	113	*SLC24A1*
6	*ARL2BP*	33	*CLRN1*	60	*IQCB1*	87	*PLK1S1*	114	*SLC7A14*
7	*ARL6*	34	*CNGA1*	61	*KCNJ13*	88	*PRCD*	115	*SNRNP200*
8	*BBIP1*	35	*CNGB1*	62	*KIF7*	89	*PROM1*	116	*SPATA7*
9	*BBS1*	36	*CRB1*	63	*KLHL7*	90	*PRPF3*	117	*TCTN1*
10	*BBS10*	37	*CRX*	64	*LCA5*	91	*PRPF31*	118	*TCTN3*
11	*BBS12*	38	*CSPP1*	65	*LRAT*	92	*PRPF4*	119	*TMEM138*
12	*BBS2*	39	*CYP4V2*	66	*LRIT3*	93	*PRPF6*	120	*TMEM216*
13	*BBS4*	40	*DFNB31*	67	*LZTFL1*	94	*PRPF8*	121	*TMEM231*
14	*BBS5*	41	*DHDDS*	68	*MAK*	95	*PRPH2*	122	*TMEM237*
15	*BBS7*	42	*EYS*	69	*MERTK*	96	*RBP3*	123	*TMEM67*
16	*BBS9*	43	*FAM161A*	70	*MKKS*	97	*RD3*	124	*TOPORS*
17	*BEST1*	44	*FLVCR1*	71	*MKS1*	98	*RDH12*	125	*TRIM32*
18	*C2orf71*	45	*FSCN2*	72	*MYO7A*	99	*RGR*	126	*TRPM1*
19	*C5orf42*	46	*GDF6*	73	*NEK2*	100	*RHO*	127	*TTC8*
20	*C8orf37*	47	*GNAT1*	74	*NMNAT1*	101	*RLBP1*	128	*TULP1*
21	*CA4*	48	*GPR179*	75	*NPHP1*	102	*ROM1*	129	*USH1C*
22	*CABP4*	49	*GPR98*	76	*NPHP4*	103	*RP1*	130	*USH1G*
23	*CACNA1F*	50	*GRK1*	77	*NR2E3*	104	*RP2*	131	*USH2A*
24	*CC2D2A*	51	*GRM6*	78	*NRL*	105	*RP9*	132	*WDPCP*
25	*CCDC28B*	52	*GUCA1B*	79	*NYX*	106	*RPE65*	133	*WFS1*
26	*CDH23*	53	*GUCY2D*	80	*OFD1*	107	*RPGR*	134	*ZNF423*
27	*CDHR1*	54	*HARS*	81	*PCDH15*	108	*RPGRIP1*	135	*ZNF513*

Supplementary Table 2: The PCR primer of the tested mutations in MYO7A

Test mutations	Forward Primers	Reverse Primers
c.5168+1G>A	TGTCCAGGCTTTCTGACTGAC	AGCACACGACCAGACAGTAGC
c.6070C>T (p.R2024X)	GTCTTGGTGTGGTGGGAAAG	AGAACCACAAGATGCACCTCC
